# Small RNA MTS1338 Configures a Stress Resistance Signature in *Mycobacterium tuberculosis*

**DOI:** 10.3390/ijms24097928

**Published:** 2023-04-27

**Authors:** Billy A. Martini, Artem S. Grigorov, Yulia V. Skvortsova, Oksana S. Bychenko, Elena G. Salina, Tatyana L. Azhikina

**Affiliations:** 1 Bach Institute of Biochemistry, Research Center of Biotechnology of the Russian Academy of Sciences, 119071 Moscow, Russia; 2 Shemyakin and Ovchinnikov Institute of Bioorganic Chemistry, Russian Academy of Sciences, 117997 Moscow, Russia

**Keywords:** small non-coding RNA, *Mycobacterium tuberculosis*, pathogen, stress resistance, survival, transcriptomic signature, RNA-seq

## Abstract

In the course of evolution, *Mycobacterium tuberculosis* (Mtb), the etiological agent of tuberculosis, has developed sophisticated strategies to evade host immune response, including the synthesis of small non-coding RNAs (sRNAs), which regulate post-transcriptional pathways involved in the stress adaptation of mycobacteria. sRNA MTS1338 is upregulated in Mtb during its infection of cultured macrophages and in the model of chronic tuberculosis, suggesting involvement in host–pathogen interactions. Here, we analyzed the role of MTS1338 in the Mtb response to macrophage-like stresses in vitro. The Mtb strain overexpressing MTS1338 demonstrated enhanced survival ability under low pH, nitrosative, and oxidative stress conditions simulating the antimicrobial environment inside macrophages. Transcriptomic analysis revealed that in MTS1338-overexpressing Mtb, the stress factors led to the activation of a number of transcriptional regulators, toxin–antitoxin modules, and stress chaperones, about half of which coincided with the genes induced in Mtb phagocytosed by macrophages. We determined the MTS1338 “core regulon”, consisting of 11 genes that were activated in all conditions under MTS1338 overexpression. Our findings indicate that MTS1338 is a stress-induced sRNA that promotes Mtb survival in macrophages by triggering adaptive transcriptional mechanisms in response to host antimicrobial defense reactions.

## 1. Introduction

Mycobacterium tuberculosis (Mtb), the causative agent of tuberculosis, is a highly adapted pathogen that evolved to persist in the human population. During latent infection (a state of continuous immune response to mycobacterial antigens without clinical symptoms), Mtb employs multiple strategies to resist and survive in an aggressive environment within the host, including hypoxia, low pH, and reactive oxygen and nitrogen species (ROS and RNS, respectively), which require effective reprogramming of bacterial metabolic pathways [1,2].

Mycobacteria are known to express small non-coding RNAs (sRNAs), which regulate mRNA and protein expression by controlling mRNA stability, processing, and access to ribosome-binding sites and which have been shown to be closely involved in bacterial survival under stressful conditions, including host immune response [3,4,5,6,7,8]. Thus, previous studies indicate that sRNA MTS1338 (ncRv11733) is strongly expressed in the stationary growth phase, under hypoxia, and during potassium limitation-induced dormancy in vitro as well as during infection in vivo [3,9,10,11]. The MTS1338 gene is located in the intergenic region between Rv1734c and Rv1733c; after transcription, the sRNA is processed to its mature form of 109 nt with two stem loops [7]. MTS1338 is associated with the dormancy survival regulator *DosR* essential for Mtb persistence [7], and is activated by NO produced in macrophages [11]. The expression of MTS1338 is induced during infection both in Mtb-resistant B6 and Mtb-susceptible I/St mouse strains, showing the highest level at week 10 post-challenge, when MTS1338 transcription in lung-residing mycobacteria is upregulated 1000-fold compared to that observed at the stationary growth phase in vitro [11]. It is noteworthy that the peak of MTS1338 expression coincides with the stage of fully developed anti-Mtb host immune response. The data, together with the fact that MTS1338 is present only in the genomes of highly pathogenic mycobacteria, suggest that this sRNA plays a role in Mtb virulence. This concept is supported by the effects caused by heterologous expression of MTS1338, which confers pathogenic properties to otherwise non-pathogenic *Mycobacterium smegmatis*, including the ability to delay phagosome maturation in macrophages, which is beneficial for the intracellular survival of the recombinant bacteria [12]. Thus, MTS1338 can be considered as an Mtb virulence factor that promotes the adaptation of mycobacteria to stressful conditions and resistance against host immune response [12].

The aim of this study was to further characterize the role of MTS1338 in Mtb infection. For this, we assessed the effects of MTS1338 overexpression on Mtb survival during macrophage infection and under macrophage-like conditions modeled in vitro. Transcriptomics of the MTS1338-overexpressing strain revealed a reproducible transcriptional signature that corresponded to that of Mtb phagocytosed by macrophages [13], suggesting the involvement of MTS1338 in the Mtb stress response and its contribution to the adaptation and survival of mycobacteria in the hostile intracellular environment.

## 2. Results

### 2.1. MTS1338-Overexpressing Mtb Strain Has Increased Viability during Macrophage Infection

**Figure 1:**
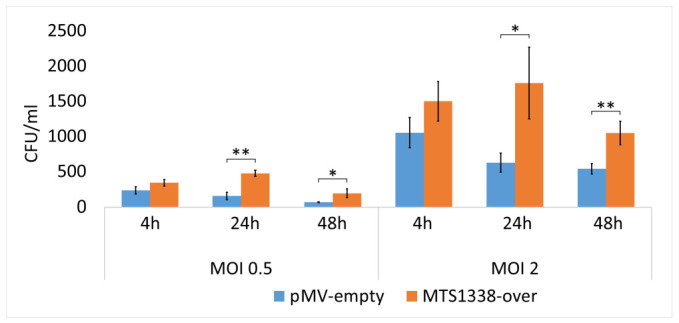
MTS1338 overexpression promotes Mtb survival in infected THP-1 cells. At 4 h post infection (p.i.), non-phagocytosed bacteria were removed, then the infected THP-1 cells were incubated for 20 h (24 h p.i.) and 44 h (48 h p.i.). CFU values were measured in triplicate and are presented as the mean ± SD; * *p* < 0.05, ** *p* < 0.01.

To analyze the biological effect of MTS1338 expression in Mtb, THP-1 cells differentiated into macrophages were infected with MTS1338-transformed (MTS1338-over) and empty vector-transformed (pMV-empty) mycobacteria in the logarithmic phase. The results revealed that the MTS1338-over strain, in which MTS1338 expression was significantly upregulated [11], had increased survival compared with the control strain at both time points (24 and 48 h after infection) and MOI values (0.5 and 2) (Figure 1), indicating enhanced resistance of MTS1338-overexpressing mycobacteria against stress conditions existing in the cytosol of infected macrophages.

### 2.2. Stress Tolerance of the MTS1338-Overexpressing Mtb Strain

To examine whether the overexpression of MTS1338 indeed protected Mtb against the hostile environment encountered in the cytosol, we subjected log-phase cultures of the MTS1338-over and pMV-empty control strains to types of stresses similar to those that mycobacteria usually face after macrophage phagocytosis: low pH, ROS, and RNS.

**Figure 2:**
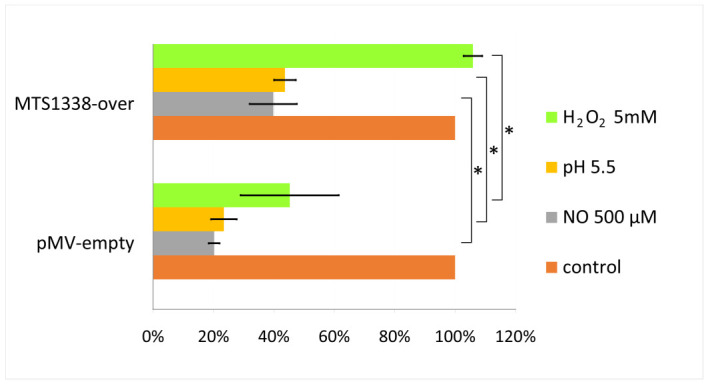
MTS1338 overexpression in Mtb promotes stress tolerance. Stress effects were measured by using [^3^H]-uracil incorporation; the viability of mycobacteria not subjected to stresses was taken as 100%. The data are presented as the mean ± SD; * *p* < 0.05.

We found that the MTS1338-over strain in the log-phase was more resistant to all the stressful conditions tested than the control strain, especially to ROS (5 mM H_2_O_2_) (Figure 2). However, for the MTS1338-over strain in the stationary phase, the maximum level of protection was observed against low pH conditions [11].

### 2.3. Transcriptome Changes Induced by MTS1338 Overexpression in the Logarithmic Growth Phase

As MTS1338 overexpression in Mtb was found to confer resistance to intra-macrophage killing and adaptation to phagocytosis-related stress factors, we speculated that transcriptome remodeling could be a reason for such survival advantages. To test this hypothesis, we performed comparative RNA-seq analysis of the MTS1338-overexpressing and control cultures in the logarithmic growth phase. The numbers of unambiguously mapped, non-chimeric fragments for each sample were between 10 and 19 million (while the number of fragments mapped to rRNA did not exceed 6% of all reads) (Table S1). DESeq2 analysis revealed 60 genes differentially expressed in the two strains, most of which (55) were upregulated, and only a few (5) were downregulated in the MTS1338-overexpressing strain. Functional analysis performed using Mycobrowser indicated that many differentially expressed genes (DEGs) encoded regulatory proteins and/or proteins strongly associated with bacterial adaptation to stress conditions, long-term persistence, and infection (Table S2). In particular, several members of the toxin–antitoxin modules, such as *vapBC* and *mazEF*, and genes encoding factors related to metal ion homeostasis, such as ArsR metalloregulatory repressors, metal sensor transcriptional regulator CmtR, and cadmium-inducible CadI, were found to be upregulated. In addition, the genes coding for the heat shock proteins Hsp and ClpB and the major secreted immunogenic protein Mpt70 were also significantly activated by MTS1338 overexpression.

### 2.4. Transcriptome Changes Induced by Macrophage-like Stresses

#### 2.4.1. Stress-Specific Transcriptional Changes

Stressful conditions affected the transcription in both Mtb strains. We found differential expression of genes associated with stress-specific metabolic pathways (according to STRING database https://string-db.org/, accessed on 16 January 2023) in the MTS1338-overexpressing (Table S3) as well as in the control (Table S4) strain.

**Figure 3:**
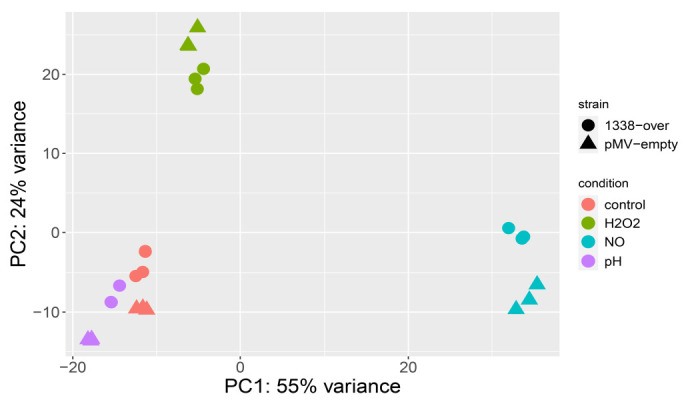
Two-component principal component analysis (PCA) of MTS1338-over (circles) and pMV-empty (triangles) samples under various conditions.

Principal component analysis (PCA) of the transcriptional profiles of the two strains revealed that the same stress factor caused similar effects in both, indicating that the influence of MTS1338 overexpression on gene transcription was less significant than that of stresses (Figure 3). Common differentially expressed genes for both the MTS1338-over and pMV empty strains after 24 h subjection to each stress factor in comparison to MTS1338-over and pMV empty logarithmic-phase liquid cultures without stress exposure are listed in Table S5.

In particular, 33 common DEGs were identified in both strains after low pH exposure, including those related to central metabolic pathways (propionate metabolism, methylcitrate cycle, and fatty acid metabolism) and those associated with general stress response, such as the *ethA*/*R* locus (Table S5). The ROS stress (H_2_O_2_) affected the expression of 90 genes, including those involved in iron starvation response (siderophore biosynthesis and siderophore-dependent iron import) and metal ion homeostasis; furthermore, genes related to stress response, such as *rsha*, *ogt*, *alka*, and Rv2191, and those involved in the interaction with the host, such as *mmpL4, mmpS4, mbtM, esxG,* and *esxH*, were found to be activated (Table S5).

Overall, 94 genes were differentially expressed in both strains after the RNS stress (NO) (Supplementary Table S5). The most characteristic change was the activation of multiple stress response genes, including *pncB2,* Rv1462, and members of the DosR regulon. Similar to H_2_O_2_, NO affected the transcription of genes regulating the response to iron starvation (siderophore biosynthesis and siderophore-dependent iron import) and metal ion homeostasis.

#### 2.4.2. MTS1338-Specific Transcriptional Response

**Figure 4:**
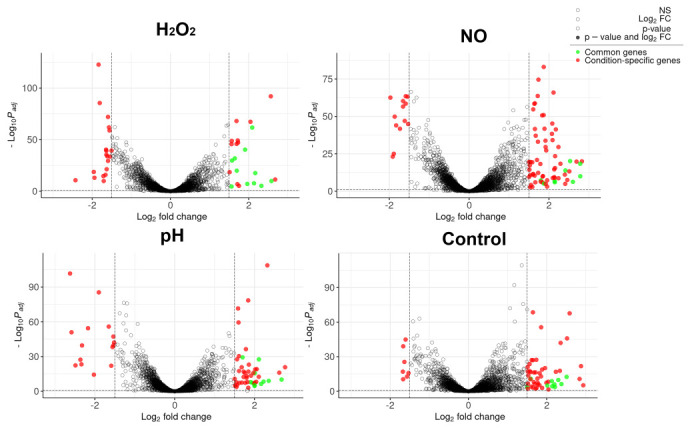
Transcriptional differences between MTS1338-overexpressing and pMV-empty strains in stressful and ‘no stress’ (control) conditions. Unfilled circles indicate genes with log_2_FC ≤ 1.5 and/or padj > 0.1, filled circles—genes with log_2_FC > 1.5 and padj < 0.1, red circles—condition-specific DEGs, and green circles—common genes in all the conditions tested.

Along with similar transcriptional responses to stress factors in the two Mtb strains, there were also differences due to MTS1338 expression (Table S6). We compared transcriptomes of MTS1338-over and pMV-empty mycobacteria in each stress condition. Overall, 42, 63, and 83 DEGs were identified in the MTS1338-over strain after H_2_O_2_, low pH, and NO exposure, respectively. The genes differentially expressed in the two strains under normal and stressful conditions are shown in Figure 4 and their functional annotation is indicated in Table 1.

**Figure 5:**
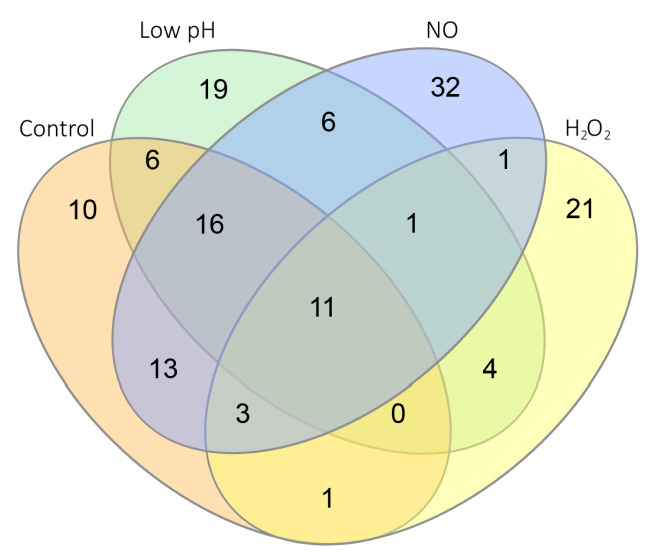
Relationships among DEGs in the MTS1338-overexpressing strain exposed to different conditions.

The distribution of genes differentially expressed in all conditions and intersecting DEGs identified in MTS1338-overexpressing mycobacteria are shown in Figure 5 and Table S6. Eleven DEGs were upregulated in the MTS1338-over strain irrespective of growth conditions (Figure 5 and Table 2), which allows us to conclude that these genes constitute a core transcriptomic signature of MTS1338 in Mtb. Thus, the overexpression of sRNA MTS1338 in mycobacteria provides a strong and stable transcriptomic response, which may be significant for Mtb survival in a hostile intracellular environment during infection.

## 3. Discussion

Bacterial sRNAs are widely recognized as powerful transcriptional regulators, allowing bacterial cells to respond quickly to changing environmental conditions [14,15]. Furthermore, in pathogenic bacterial species, including Mtb, sRNAs may act as virulence factors, promoting survival in the infected host [16,17,18]. Thus, in Mtb, the synthesis of sRNAs and their targets is upregulated after mycobacteria are phagocytosed by alveolar macrophages, where they can reside for a long time in a dormant state, resisting harsh intracellular conditions such as high acidity and oxidative stress [19].

MTS1338, a highly conserved sRNA present exclusively in the genomes of pathogenic mycobacteria, is significantly accumulated in dormant Mtb cells and upregulated in mycobacteria phagocytosed by macrophages in vitro and in mouse models of infection [10,20,21], suggesting the involvement of MTS1338 in host–pathogen interactions. In our previous work [11], we used the mouse model of infection, and demonstrated that MTS1338 is upregulated at the start of infection and remains at a high level until mouse death. We analyzed the MTS1338-overexpressing Mtb strain in the stationary phase and observed growth retardation and transcriptional shifts consistent with decreased bacterial metabolism, translational activity, and cell division [11] characteristic for a general survival strategy in bacteria [22]. It should be noted that the transcriptome of the MTS1338-overexpressing strain in the logarithmic phase obtained in the present study (Table S2) significantly differs from that in the stationary phase [11], reflecting the primary changes caused by MTS1338 overexpression.

MTS1338-overexpressing Mtb in the stationary phase is more resistant to low pH than the control (empty vector) strain [11], but the stress-protective effects of MTS1338 overexpression in the logarithmic phase appear to be more pronounced. While in the stationary cultures, the effects of H_2_O_2_ and NO on the Mtb metabolic activity are negligible, in the logarithmic cultures, they are significant (Figure 2).

Considering that mycobacteria phagocytosed by macrophages could be mainly in the active growth state [19,23,24], in this study, we investigated how MTS1338-overexpressing mycobacteria in the logarithmic phase responded to stress factors similar to those encountered by Mtb in macrophages.

The results indicated that MTS1338-overexpressing Mtb could better resist macrophage-like stresses such as low pH, ROS, and RNS, as evidenced by survival analysis, suggesting a role of MTS1338 in Mtb adaptation to the intracellular environment. Comparative transcriptional profiling of the MTS1338-over and pMV-empty strains subjected to stress conditions for 24 h revealed that although MTS1338 influence on Mtb transcriptional activity was not as strong as that of stress factors (Figure 3), there were also significant differences in the stress response between the two strains due to MTS1338 overexpression (Table 1).

Since macrophages are the first line of defense against mycobacterial infection, we compared the Mtb transcriptomic profiles obtained here with those observed in peritoneal macrophages of I/St mice [13], and found that about 50% of the DEGs identified in the stressed MTS1338-overexpressing strain in vitro overlapped with those activated in Mtb residing in peritoneal macrophages: 32 of 60 DEGs in normal conditions, 32 of 63 at low pH, 16 of 42 with ROS, and 54 of 83 with RNS. These results suggest that the upregulation of MTS1338 during Mtb infection triggers the adaptive mechanisms that support mycobacterial survival in the aggressive intra-macrophage environment.

We also identified 11 common DEGs that were upregulated in all (normal and stress) conditions and, therefore, could be considered as a core transcriptomic signature of MTS1338-over. Among them are:

- *mpt70*, encoding a secreted immunodominant antigen, Mpt70, which is upregulated in response to IFN-γ and nutrient or oxygen deprivation of infected macrophages [25,26] and is known to promote T cell differentiation [27]. Mpt70 is mainly expressed at the later stages of in vivo infection and could be functionally linked to the genes involved in Mtb persistence, such as *DosR* [27].
- Rv2034, encoding the ArsR repressor, which senses heavy metal ions and causes derepression of stress-induced operons. In Mtb, Rv2034 upregulates *phoP* and *dosR* genes [28,29]. PhoP controls the expression of Mtb key virulence factors such as ESX-1, cell wall components, and Ag85 antigen via sRNA Mcr7 and messenger metabolite c-di-AMP, and is essential for Mtb virulence and persistence [8,30]; furthermore, it plays a major role in Mtb survival under hypoxia and acidic pH [31,32]. DosR, a key player in Mtb adaptation to non-replicating survival in the hypoxic environment [22,33], acts together with PhoP in sensing macrophage-like stresses [32]. However, although MTS1338 is regulated by DosR, it is not fully DosR-dependent [7], and neither *dosR* nor any other Dos-regulated genes were differentially expressed in the MTS1338-over strain. Similarly, the PhoP-encoding gene (Rv0757) also was not identified as a DEG in this study.
- Rv2035, together with Rv2034, is predicted to constitute a novel toxin–antitoxin system involved in Mtb latency during macrophage infection [34].
- *cmtR* (Rv1994c), a member of the ArsR family, functions as a redox sensor and can be significantly activated by H_2_O_2_ stress [35]. CmtR can physically interact with the negative regulator Zur and de-repress the expression of the *esx-3* operon, leading to Zn^2+^ accumulation and the promotion of ROS detoxification in mycobacteria [35]. Consequently, CmtR contributes to bacterial survival in macrophages and in the lungs of infected mice [35].
- The Rv2641–Rv2642 operon. Rv2641 (*cadI*) encodes a cadmium-induced protein [36] that could be also sensitive to copper and zinc in *Mycobacterium bovis BCG* [37,38]. The function of CadI is similar to that of metallothioneins—low-molecular-weight cysteine-rich proteins that protect Mtb against metal toxicity [37]. Rv2642 belongs to the ArsR family, and regulates *cadI* and its own expression through binding to a conserved motif [39]. Rv2642 and Rv2643 are immunogenic and induce significant IFN-γ response in individuals with latent tuberculosis infection [40].

We speculate that the 11 DEGs activated in the MTS1338-overexpressing strain irrespective of growth conditions could be under the direct regulation of MTS1338, representing a mechanism evolved in Mtb to support its survival in the host during infection.

## 4. Materials and Methods

### 4.1. MTS1338-Overexpressing Strain

The MTS1338 gene-containing vector was constructed on the basis of pMV261 [41] as previously described [10], and used to transform the Mtb H37Rv strain through electroporation. MTS1338 overexpression was confirmed using qRT-PCR [10]. Mycobacteria transformed with an empty pMV261 vector were used as control.

### 4.2. Bacterial Growth Conditions

The MTS1338-overexpressing and control Mtb strains were recovered from frozen stocks through incubation in Sauton medium containing (per liter) 0.5 g KH_2_PO_4_, 1.4 g MgSO_4_ × 7 H_2_O, 4 g L-asparagine, 60 mL glycerol, 0.05 g ferric ammonium citrate, 2 g sodium citrate, and 0.1 mL 1% ZnSO_4_ (pH 7.0 adjusted with 1 M NaOH) and supplemented with 10% ADC growth supplement [42], 0.05% Tween 80 (Panreac, Barcelona, Spain), and 50 µg/mL kanamycin (Sigma-Aldrich, St. Louis, MO, USA) (full medium). After growth for 10 days at 37 °C with agitation (200 rpm), the starter cultures were inoculated into fresh medium, grown until the log phase, and used for stress survival and RNA-seq experiments.

### 4.3. Stress Effects on Mtb Survival

To induce low pH stress, recombinant bacteria in the log phase (OD_600_ = 1) were collected, washed with PBS, diluted to OD_600_ = 0.2 (10^7^ CFU/mL) in full Sauton medium, pH 5.5, and incubated for 48 h. For nitrosative or oxidative stress, the bacteria (10^7^ CFU/mL) were incubated for 48 h in full Sauton medium (pH 7.0) supplemented with 0.5 mM DETA NONOate (Sigma-Aldrich, St. Louis, MO, USA) or 10 mM H_2_O_2_ (Fluka Chemie AG, Buch, Germany), respectively. For the no-stress control, the strains were grown in Sauton medium. The inhibitory effects were estimated using the level of [^3^H]-uracil incorporation, known to directly correlate (ρ > 99) with CFU counts in mycobacterial cultures [43,44].

### 4.4. [^3^H]-Uracil Incorporation

Culture samples (1 mL) were incubated with 1 μL of 5.6-^3^H uracil (1 mCi) at 37 °C with agitation for 20 h. Then, 200 μL of each sample was mixed with 3 mL of 7% ice-cold CCl_3_COOH, incubated at 4 °C for 15 min, and filtered through glass microfiber filters (Whatman, Inc., Clifton, NJ, USA), which were subsequently washed with 3 mL of 7% CCl_3_COOH and 6 mL of 96% ethanol, and placed into 10 mL of scintillation mixture (Ultima Gold AB; PerkinElmer, Waltham, MA, USA). Radioactivity was determined using an LS analyzer ((Beckman Instruments Inc., Palo Alto, CA, USA) and expressed as counts per min (cpm).

### 4.5. RNA Isolation

Bacterial cultures exposed to stressful conditions for 24 h were rapidly cooled on ice, centrifuged, and subjected to disruption with 0.1 mm zirconia beads in a Bead Beater homogenizer (BioSpec Products, Bartlesville, OK, USA) as previously described [45]. Total RNA was isolated through phenol-chloroform extraction, treated with Turbo DNase (Life Technologies, Gaithersburg, MD, USA) to remove traces of genomic DNA, purified with the RNeasy Mini Kit (Qiagen, Venlo, The Netherlands), and analyzed for quality and quantity through spectrophotometry; RNA integrity was assessed in 1% agarose gels.

### 4.6. RNA-Seq and Data Analysis

RNA samples were depleted of 16S and 23S rRNA as previously described [46] using ribonuclease H (NEB, USA) and custom oligodeoxyribonucleotides 16S_F: /5′Phos/ 5′-AGAGTTTGATCCTGGCTCAG-3′, 16S_R: 5′-AAGGAGGTGATCCAGCCGCA-3′, 23S_F: /5′Phos/ 5′-YGGTGGATGCCTTGGC-3′, and 23S_R: 5′-YRCTTAGATGCTTTCAGCRBTTATC-3′ (DNA Synthesis, Moscow, Russia); (Y stands for pyrimidine (C or T), R for purine (A or G), and B for C, G, or T (not A). Sequencing libraries were generated using the resulting ribosomal transcript-depleted RNA and the NEBNext Ultra II Directional RNA Library Prep Kit (NEB) according to the manufacturer’s protocol. Sequencing was performed in an Illumina NovaSeq 6000 in paired-end mode as 150 nt long reads. After quality control evaluation and trimming of poor-quality reads, the remaining reads were mapped on the reference Mtb genome (AL123456.3, http://www.ncbi.nlm.nih.gov/, accessed on 2 May 2022) using Bowtie2 [47]. The alignment was performed with the “-local” option, which allows leaving 5′ and 3′ ends unaligned, and the mapped reads for all genes were calculated using functions of the featureCounts package [48] built into the author’s script. DEGs were identified using the software package DESeq2 [49] according to the following criteria: adjusted *p*-value < 0.1 and log2 fold change (log2FC) value ≥ 1.5. The distribution of DEGs according to functional categories was performed using the Mycobrowser database (https://mycobrowser.epfl.ch/, accessed on 16 May 2022). All RNA-seq data generated in this study have been deposited in the GEO repository under accession number GSE218354.

### 4.7. Mtb Infection of Macrophages

THP-1 human monocyte cells (ATCC #TIB-202) were seeded on 24-well plates (Costar, USA) and differentiated in the presence of 100 ng/mL phorbol-12-meristat-13-acetate (FMA, Sigma-Aldrich, St. Louis, MO, USA) in RPMI-1640 medium (Gibco BRL, Gaithersburg, MD, USA) supplemented with 10% fetal bovine serum (FCS; Gibco BRL, Gaithersburg, MD, USA) at 37 °C in a 5% CO_2_ incubator for 24 h until 70~80% confluence. For infection, the differentiated THP-1 cells were collected and seeded on 24-well plates (5 × 10^4^ cells/well); then, mycobacteria, which had been grown to OD_600_ = 0.8–1.0, washed with PBS, and resuspended in RPMI-1640/10% FCS, were added to cells at a multiplicity of infection (MOI) of 0.5:1 and 2:1. For each strain, equal numbers of bacteria (according to OD) were used for infection. After incubation for 4 h, the infected cell monolayers were washed three times with PBS, cultured for 0, 24, and 48 h, washed again with PBS, lysed in ice-cold 0.01% sodium dodecyl sulphate, and 10-fold serially diluted in milliQ water with 0.05% Tween-80. Then, 10 µL of each dilution was spread on Sauton agar plates in triplicate, and the number of colonies was counted after 21 days to determine CFU/mL values.

### 4.8. Statistical Analysis

The results are expressed as the mean ± standard deviation of three independent experiments; *p* < 0.05 was considered to indicate statistically significant differences. Statistical analysis was performed using GraphPad Prism 6 (GraphPad Software, La Jolla, CA, USA).

## 5. Conclusions

Our results indicate that sRNA MTS1338 promoted Mtb survival in infected macrophages and increased its resistance to macrophage-like stresses in vitro. Transcriptomic profiling showed that in all conditions, MTS1338 activated 11 common genes associated with transcriptional regulation, metal and redox sensing, and the toxin–antitoxin system involved in Mtb latency during macrophage infection, suggesting that this set of MTS1338-dependent ‘core’ genes could constitute a system of Mtb adaptation to any adverse conditions. We suggest that MTS1338 regulates Mtb gene expression in a multi-target manner. At the same time, MTS1338 elicited distinct transcriptional responses to different types of stresses (low pH, ROS, RNS), thus providing Mtb with stress-specific resistance mechanisms. Our findings support the notion that MTS1338 could be an Mtb virulence factor supporting pathogen survival and long-term persistence in the infected host.

## Figures and Tables

**Table 1:** Functional distribution of DEGs in the MTS1338-overexpressing strain exposed to macrophage-like stresses.

Stress Factor	Functional Category According to Mycobrowser (https://mycobrowser.epfl.ch/, accessed on 16 May 2022)	Genes	Up/Down
pH	Regulatory proteins	Rv0260c	↓
		Rv0792c Rv1395 *furA* Rv1990c *cmtR* Rv2034 Rv2250c Rv2642 Rv3183 Rv3334	↑
	Information pathways	Rv0516c Rv2464c	↑
	Cell wall and cell processes	*iniB* Rv0841 *arsC mpt70*	↑
		*esxK esxL rocE*	↓
	Intermediary metabolism and respiration	*glbN nirB nirD* Rv3741c Rv3742c	↓
		*cyp138 galK* Rv0793 *grcC2 frdA* Rv1990A	↑
	Lipid metabolism	*papA4*	↑
	PE/PPE	*pe13 ppe18 ppe19 ppe43 pe27*	↓
		*ppe29 ppe37*	↑
	Virulence, detoxification, adaptation	*hsp vapB30 vapC30 higB mazE6 mazF6*	↑
	Insertion seqs and phages	Rv3428c	↑
	Conserved hypotheticals (unknown function)	Rv1954A Rv0140 Rv0142 Rv0826 Rv1044 Rv1357c Rv1989c Rv1993c Rv2035 Rv2466c *cadI* Rv3054c Rv3182 Rv3188 Rv3189 Rv3659c	↑
		Rv0259c	↓
H_2_O_2_	Regulatory proteins	Rv0260c	↓
		Rv1129c Rv1395 Rv1473A *cmtR* Rv0792c Rv2034 Rv2642 *whiB6*	↑
	Information pathways	Rv2464c	↑
		Rv3201c	↓
	Cell wall and cell processes	Rv0188 Rv2617c Rv3289c	↓
		Rv1473 *mpt70*	↑
	Intermediary metabolism and respiration	*galK* Rv0793 *prpD prpC*	↑
		Rv1279 *glbN lat*	↓
	Lipid metabolism	*pks11 desA3* Rv3371	↓
	Pe/ppe	*ppe27*	↑
		*pe20 ppe31*	↓
	Virulence, detoxification, adaptation	Rv3660c	↑
	Insertion seqs and phages	Rv0095c Rv0829	↓
	Conserved hypotheticals (unknown function)	Rv0259c Rv2015c Rv1278 Rv1765c	↓
		Rv0791c Rv0826 *cadI* Rv3659c Rv2035 Rv2422	↑
NO	Regulatory proteins	Rv0196 Rv0792c *kmtR csoR* Rv1219c Rv1395 Rv1674c *furA* Rv1990c *cmtR* Rv2034 Rv2250c Rv2642 Rv2989 Rv3183 Rv3334 Rv3840 *whiB6*	↑
	Cell wall and cell processes	*lprK*	↓
		Rv0841 *arsC mpt70* Rv2963 Rv3657c	↑
	Intermediary metabolism and respiration	*bioF2 lipF*	↓
		*galK* Rv0793 *cysD hisE* Rv2250A *ethA*	↑
	Lipid metabolism	*acpA fadD34*	↓
		Rv0830 *papA4 lipX*	↑
	PE/PPE	*pe15 ppe20 ppe29 pe20 ppe31 ppe37*	↑
		*pe31*	↓
	Virulence, detoxification, adaptation	*mce1A mce1B mce1D*	↓
		*mymT hsp clpB vapB30 vapC30 yrbE3A mazE6 mazF6* Rv3660c	↑
	Insertion seqs and phages	Rv2013 Rv3428c Rv3751	↑
	Conserved hypotheticals (unknown function)	Rv0034 Rv1157c Rv1158c Rv1697	↓
		Rv0448c Rv0724A Rv0826 Rv0968 Rv1044 Rv1048c Rv1989c Rv1995 Rv2016 Rv2035 Rv2036 Rv2327 *cadI* Rv2662 Rv3054c Rv3182 Rv3188 Rv3189 Rv3659c Rv3839	↑

**Table 2:** A core transcriptomic signature of sRNA MTS1338 in Mtb, comprising DEGs upregulated by MTS1338 overexpression under normal and stressful conditions (log_2_FC > 1.5 and padj < 0.1).

Gene	Product	Log_2_FC			
		Ctrl	pH	H_2_O_2_	NO
*galK*	Probable galactokinase GalK (galactose kinase)	1.50	2.00	2.14	2.15
Rv0792c	Probable transcriptional regulatory protein	1.59	1.70	1.97	1.80
Rv0826	Conserved hypothetical protein	2.38	1.95	2.32	2.60
Rv1395	Transcriptional regulatory protein	2.30	2.01	1.65	2.53
*cmtR*	Metal sensor transcriptional regulator CmtR (ArsR-SmtB family)	2.14	2.17	1.58	1.88
Rv2034	ArsR repressor protein	3.08	2.68	2.58	3.45
Rv2035	Conserved hypothetical protein	2.13	1.90	1.56	2.41
*cadI*	Cadmium-inducible protein CadI	2.21	2.00	2.17	3.26
Rv2642	Possible transcriptional regulatory protein	2.20	2.23	1.91	2.79
*mpt70*	Major secreted immunogenic protein Mpt70	2.51	2.36	2.09	2.78

## Data Availability

All RNA-seq data generated for this study have been deposited in the GEO repository under accession number GSE218354.

## Supplementary Materials

The supporting information can be downloaded at: https://www.mdpi.com/article/10.3390/ijms24097928/s1.
